# COVID-19 Mortality in the Delta Wave in India: A Hospital-Based Study From Ramanagara District, Karnataka

**DOI:** 10.7759/cureus.43678

**Published:** 2023-08-18

**Authors:** Amita Mukhopadhyay, Geetha KB, Ipsita Debata, Charithra BV, Manju Prakash, Tejas J

**Affiliations:** 1 Community Medicine, Institute of Health Management Research Bangalore (IHMR-B), Bangalore, IND; 2 Forensic Medicine, Dr. Chandramma Dayananda Sagar Institute of Medical Education and Research, Bangalore, IND; 3 Community Medicine, Kalinga Institute of Medical Sciences, Bhubaneswar, IND; 4 Anesthesiology, DM Wayanand Institute of Medical Sciences, Wayanad, IND; 5 Forensic Medicine, Karpaga Vinayaga Institute of Medical Sciences and Research Center, Maduranthakam, IND

**Keywords:** covid-19, sars cov-2, delta variant, second wave, covid-19 epidemiology, inhospital mortality

## Abstract

Introduction: Coronavirus 19 (COVID-19) disease spread rapidly over the world since its inception in December 2019 in Wuhan, China. India too was crippled by the burden of high caseloads and deaths. The first death caused by COVID-19 in Karnataka was reported on March 13, 2020. There is a plethora of information on the descriptive statistics, epidemiology, and management of COVID-19 cases. However, there has not been an in-depth and extensive exploration of COVID-19 mortality data in terms of published research from India. The study area was a 300 bedded tertiary care center in Ramnagara district, Karnataka. During the second wave, 150 beds were dedicated to COVID-19 cases referred from government centers. This study was carried out to assess the in-hospital mortality at this institute during the second wave. The expected outcome of this study was to shed light on co-morbidities associated with mortality, the age and sex distribution in mortality, and any other significant factors influencing mortality due to COVID-19.

Methodology: A hospital-based, retrospective, and observational-analytical study was carried out during April-August 2021, the second wave of COVID-19. The data included all deaths recorded in-hospital during the dedicated COVID-19 referral center status. Data were collected from case sheets and mortality audit forms that included basic demographics, symptoms, co-morbidities, admission pathway, transfer to ICU, oxygen therapy, ventilator requirement, duration of hospitalization, laboratory profile, and management modalities prior to death. Data were compiled into Microsoft Excel and were analyzed with JASP software (open source). Data were interpreted in terms of frequencies, averages with standard deviation, and bivariate and multivariate analysis.

Results: We analyzed mortality audits of 91 adult patients and one neonate. The male-to-female ratio was 1.67:1 (> 60% male), with an average age of 53.4 years (standard deviation 15.4 years). Most of the patients fell in the age range of 36 to 65 years (65%). The average duration was 5.6 days (range 0-35 days). The most common symptom was fever (84, 92.31%), followed by breathlessness (77, 84.62%) and fatigue (65, 71.43%). Only 10 had a positive contact history and only one patient reported travel to a containment zone. The source of infection was indeterminate in the majority of cases. Diabetes mellitus and hypertension were the commonest associated comorbidities. Almost three-quarters of the patients were tachypneic at admission and nearly 90% had low levels which included 43 patients with critically low SpO_2_. The inflammatory indicators, such as WBC count, CRP, and d-dimer, were raised in many patients (WBC count raised in 40% and d-dimer, CRP raised in > 50% of cases). A striking 83% of the patients had hyperglycemia. The most common immediate cause of death pertained to the respiratory system (ARDS, refractory hypoxia, respiratory) in more than half of the patients.

Conclusion: This study reported the clinical and laboratory characteristics of 91 adult COVID-19 mortality cases at a teaching hospital at the peak of the Delta wave in Karnataka. While inflammatory indicators such as WBC count, CRP, and d-dimer were raised in many patients, our most remarkable finding was the high frequency of hyperglycemia. The findings of our study would contribute to enhancing the understanding of the clinical correlates and progression of COVID-19.

## Introduction

Coronavirus disease-19 (COVID-19) disease rapidly attained pandemic status since its inception in December 2019 in Wuhan, China [1]. As per WHO data, there were more than two hundred million active cases and four million deaths due to COVID-19 reported worldwide by August 2021 when the peak of the Delta wave was subsiding [2]. At the same time, India had recorded more than 30 million active cases and more than 430,000 deaths, with more than 37,000 deaths reported from Karnataka state since the commencement of the first wave in January-March 2020 [3]. After a brief decline in cases between November 2020 and February 2021, India encountered the challenge of the second wave of COVID-19 (also known as the Delta wave after the prevalent strain of the virus) with close to 20 million cases and a quarter million deaths recorded in a four-month period spanning April to July 2021 [4]. Before the second wave began, India accounted for 16% of the global burden of COVID-19 cases. It was the second worst affected country in terms of cumulative case total and third in terms of absolute number of recorded deaths due to COVID-19 [5,6]. The overall case fatality rate in India was 1.2%, which was the lowest among the top 20 worst-affected countries [7]. The first COVID-19 death in Karnataka was reported on March 13, 2020 [8]. There is a plethora of information on the descriptive statistics, epidemiology, and management of COVID-19 cases. Mortality studies focus on large-scale data from states, districts, etc., based on government reports and case fatality rate statistics. However, there has not been an extensive exploration of the clinical and demographic correlates of COVID-19 mortality in terms of published research from India. The authors of this paper have worked as part of the COVID-19 emergency response team in a 300 bedded tertiary care center in Ramnagara district, Karnataka. During the second wave starting in April 2021, following a government mandate, routine services were suspended and the hospital was converted to an exclusive COVID-19 referral facility with 150 beds dedicated to COVID-19 cases referred from government centers. This hospital has cared for some of the most serious and moribund patients, including conducting vaginal and caesarean delivery procedures for pregnant women diagnosed with COVID-19 for whom this hospital was the only facility in the district offering perinatal care services. Therefore, we proposed to carry out a study with the objectives to evaluate in-hospital mortality due to COVID-19 during the second wave in terms of (a) demographic and clinicopathological features of patients who died after being diagnosed with COVID-19 and referred to this hospital (in-hospital deaths) and (b) analysis of clinical and laboratory associations of COVID-19 mortality.

## Materials and methods

A hospital-based, retrospective, and observational-analytical study was carried out by the Department of Community Medicine and Forensic Medicine at Dr. Chandramma Dayananda Sagar Institute of Medical Sciences and Research Center, during April-August 2021, the second COVID-19 wave. Scientific and ethical approval was obtained from the Institutional Ethics Committee (Approval number: CDSIMER/MR/0025/IEC/2021) of the tertiary hospital and attached medical teaching institute. Permission was taken from the Head of the Institute. All data were anonymized and shared only with authorized personnel. Due to restrictions on sharing, we have not attached the data files. However, anonymized data may be shared with researchers upon reasonable request. The sample included all deaths recorded in-hospital during the dedicated COVID-19 referral center status. Data were collected from case sheets and mortality audit forms which were conducted and recorded by the Department of Forensic Medicine.

Inclusion criteria

All mortality cases that satisfy the following criteria: RAT/RTPCR positive for SARS-CoV-2, clinically diagnosed COVID-19/COVID-19-like syndrome/SARI, all mortality cases having complete and extensive records available for data extraction.

Exclusion criteria

Patient dead on arrival, death within 24 hours of admission, and medico-legal cases. Data were sourced from case sheets and mortality audits. The extracted data included basic demographics (age and gender), symptoms, co-morbidities, admission pathway (ward or ICU), transfer to ICU, oxygen therapy, ventilator requirement, duration of hospitalization, laboratory profile, and management modalities before death. We used Microsoft Excel to compile the data and JASP software (open source) for analysis [9]. Data were interpreted in terms of frequencies, averages with standard deviation, and bivariate and multivariate analysis. A p-value < 0.05 was considered statistically significant.

## Results

**Figure 1:**
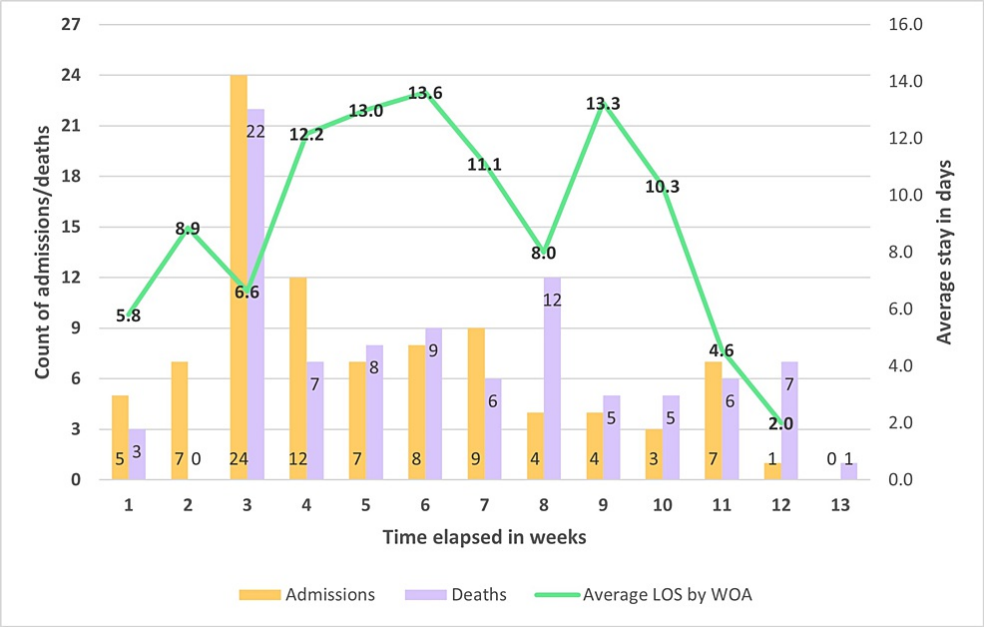
Admissions, deaths, and average length of stay (week 1 to week 13) LOS: Length of stay; WOA: Week of admission

We studied the mortality audits of 91 adult patients and one neonate. The adult patients’ averages are presented in the result tables, while the baby’s clinical and laboratory parameters are detailed separately. The first referral case was admitted to the hospital on April 15, 2021, and the weeks were numbered accordingly. Week 3 had the highest number of admissions and deaths, with 24 admissions and 22 deaths. The highest number of admissions (10) was observed on April 30, 2021. The average length of stay (ALOS) was calculated, and the range for the duration of hospitalization was 36 days (0-36 days), whereas the average duration of hospitalization preceding death was 9.3 days (F 10.0, M 8.9). The oldest age group had the lowest duration (average 6.22 days), while the 56-65 years age group had the highest duration (average 13.1 days). The timeline of mortality and mortality is depicted in Figure 1.

The 91 adults in the study sample had a male-to-female ratio of 1.67:1 (> 60% male), with an average age of 53.4 years (standard deviation 15.4 years). Most of the patients fell in the age range of 36 to 65 years (around 65%). The age-sex distribution of adult mortalities has been listed in Table 1.

**Table 1:** Age-sex distribution of adult mortalities Note: While “Female” and “Male” sections show row percentages to illustrate sex distribution in age bins, the "Total” section shows column percentage.

	Female		Male		Total	
Age group	n	%	n	%	n	%
26 – 35	3	30.0	7	70.0	10	11.0
36 – 45	4	20.0	16	80.0	20	22.0
46 – 55	11	50.0	11	50.0	22	24.2
56 – 65	10	58.8	7	41.2	17	18.7
66 – 75	3	23.1	10	76.9	13	14.3
76 – 99	3	33.3	6	66.7	9	9.9
Total	34	37.4	57	62.6	91	100.0

Duration of symptoms preceding admission was available for 90 of the patients. The average duration was 5.6 days (range 0-35 days). The most common symptom was fever, followed by breathlessness, fatigue, cough, and sputum production. The symptoms have been listed in Table 2.

**Table 2:** Common reported symptoms

Symptom	Yes		No		Total
	n	%	n	%	N
Fever	84	92.31	7	7.69	91
Breathlessness	77	84.62	14	15.38	91
Fatigue	65	71.43	26	28.57	91
Cough	53	58.24	38	41.76	91
Sputum production	29	31.87	62	68.13	91

The majority of the patients reported no conclusive history indicating the source of infection. Among the 91 adult patients, only ten had a positive contact history and only one patient reported travel to a containment zone. The source of infection was indeterminate in the majority of cases. Table 3 shows the possible source of infection.

**Table 3:** Possible source of infection

Variable		Frequency	Percentage
History of contact with positive case	Yes	10	11
	No	81	89
History of travel to containment zone	Yes	1	1.1
	No	90	98.9
History of Covid-19 case in family	Yes	5	5.5
	No	86	94.5

Diabetes mellitus and hypertension were the most commonly associated comorbidities, as depicted in Table 4. Men and women did not differ significantly in terms of the proportion of diabetics and hypertensives in each sex. Only three patients gave a positive smoking history, and nine reported alcohol consumption.

**Table 4:** Commonly observed comorbidities Note: No significant difference observed in sex distribution of comorbidities

Comorbidity		Frequency	Percentage	P value for sex difference
Diabetes Mellitus	Yes	32	35.2	0.4
	No	59	64.8	
Hypertension	Yes	30	33.0	0.2
	No	61	67.0	
Cerebro Vascular Disease/Stroke	Yes	5	5.5	0.9
	No	86	94.5	
Lung Disease	Yes	3	3.3	0.3
	No	88	96.7	
Chronic Renal Disease	Yes	1	1.1	0.4
	No	90	98.9	
Immunocompromised	Yes	2	2.2	0.3
	No	89	97.8	

Close to three-quarters of the patients were tachypneic at admission and nearly 90% had low levels, including 43 patients with critically low SpO2. We noted that while 45% of the patients had tachycardia, only about five percent were febrile at admission. Approximately a fifth of the patients had a high systolic BP. Diastolic BP was within normal limits in all except for six patients. Three patients were found to be hypotensive at admission. There were no significant sex differences in physiological variables. The vitals at the time of admission have been depicted in Table 5.

**Table 5:** Vital signs at admission

Variable	Frequency	Percentage
Respiratory Rate (per min)		
18 - 23	25	27.5
24 - 29	37	40.7
≥ 30	29	31.9
SpO2		
> 94	11	12.1
85 - 94	37	40.7
60 - 84	32	35.2
< 60	11	12.1
Pulse Rate (per min)		
< 60	2	2.2
60 - 100	48	52.7
> 100	41	45.1
Temperature (Fahrenheit)		
< 97	6	6.6
97 - 99	80	87.9
> 99	5	5.5
Systolic BP		
< 90	2	2.2
90 - 119	37	40.7
120 - 139	31	34.1
≥ 140	21	23.1
Diastolic BP		
< 60	1	1.1
60 - 79	36	39.6
80 - 99	49	53.8
≥ 100	5	5.5

The ranges and averages of the laboratory parameters are shown in Table 6. Values did not differ significantly by sex or age.

**Table 6:** Laboratory parameters: Descriptives

Parameter	Sex	n	Mean	SD	Min	Max
Hemoglobin (gm / dl)	F	30	12.7	1.9	8.7	17.7
	M	44	14.3	2.6	3.2	19
	T	74	13.7	2.5	3.2	19
Leucocytes (count / cubic mm)	F	29	11403.1	5951.1	2800	22620
	M	45	10024.9	6406.3	410	27660
	T	74	10565	6227.4	410	27660
Platelets (count / cubic mm)	F	30	234833.3	83761.2	77000	459000
	M	44	195090.9	86288.8	32000	430000
	T	74	211202.7	86942.3	32000	459000
Urea (mg / dl)	F	33	47.4	27.6	8	123
	M	47	52.9	39.6	13	230.6
	T	80	50.6	35.1	8	230.6
Creatinine mg / dl	F	33	0.9	0.5	0.3	2.8
	M	49	1.2	0.9	0.3	4.3
	T	82	1.1	0.8	0.3	4.3
D-dimer (ng / ml)	F	31	1738.6	1685	81	4000
	M	45	1474.9	1559.3	127.3	4000
	T	76	1582.5	1606	81	4000
C Reactive Protein (mg / l)	F	32	12.4	7.5	1.3	27.6
	M	47	12	9.9	0.7	40.5
	T	79	12.2	9	0.7	40.5
Bilirubin (mg / dl)	F	31	0.5	0.2	0.2	1.2
	M	48	1.1	1.5	0.3	10.6
	T	79	0.9	1.2	0.2	10.6
Alkaline Phosphatase Units / L	F	32	103.5	42.8	52	197
	M	46	101.2	43.2	47	242
	T	78	102.1	42.8	47	242
Gamma glutamyl transferase Units / L	F	30	119.8	75	20	312
	M	41	108.9	113.2	19	505
	T	71	113.5	98.4	19	505
Blood glucose (mg / dl)	F	27	248.6	124.7	94	628
	M	38	286.1	147.8	83	644
	T	65	270.5	138.9	83	644

Table 7 shows the distribution of these parameters by normal and pathological range. We found that inflammatory indicators such as WBC count, CRP, and d-dimer were raised in many patients (WBC count raised in nearly 40% and d-dimer, CRP raised in > 50% of cases). A striking 83% of the patients had hyperglycemia whereas 35% were known diabetics. Blood urea was also raised in four-fifths of the cases with high creatinine seen in more than a fifth. We also raised GGT in 80% of the cases, however, ALP levels were normal in most of them.

**Table 7:** Laboratory parameters by normal and pathological values Note: There were no women with raised bilirubin values in the study sample.

Variable	Frequency	Percentage	P value for sex difference
Hemoglobin (gm/dL)			
Low (F: <12.0 M: <13.0)	20	27.0	0.6
Normal	47	63.5	
Raised (F: >15.0; M: >17.0)	7	9.5	
Leucocytes (count/cubic mm)			
Low (<4500)	9	12.2	0.9
Normal	37	50.0	
Raised (>11000)	28	37.8	
Platelets (count/cubic mm)			
<150000	18	24.3	0.1
150000-450000	55	74.3	
>450000	1	1.4	
C Reactive Protein (mg/L)			
Normal (<10)	33	41.8	0.3
Raised	46	58.2	
D-dimer (ng/ml)			
Normal (<500)	34	44.7	1.0
Raised	42	55.3	
Blood glucose (mg/dL)			
Normal (70-140)	11	16.9	0.3
Raised	54	83.1	
Creatinine (mg/dL)			
Low (F: <0.5 M: <0.6)	8	9.8	0.4
Normal	55	67.1	
Raised (F: >1.2; M: >1.3)	19	23.2	
Urea (mg/dL)			
Normal (7-25)	14	17.5	0.9
Raised	66	82.5	
Bilirubin (mg/dL)			
0.2-1.2	73	92.4	0.04
>1.2	6	7.6	
Alkaline Phosphatase Units/L			
Normal (44-147)	69	88.5	0.3
Raised	9	11.5	
Gamma glutamyl transferase Units/L			
Normal (5-40)	14	19.7	0.2
Raised	57	80.3	

In our study, the immediate cause of death as noted by the clinician certifying death pertained to the respiratory system (ARDS, refractory hypoxia, respiratory) in more than half of the patients. Cardiac causes such as acute coronary syndrome and cardiac arrest were the next commonest cause of death followed by septic shock. Acute kidney injury contributed to only 5% of deaths despite abnormal renal parameters in 80% of the patients. There was only one neonate born to a COVID-19-positive mother. The hospital was the only facility in the district where advanced maternity care was provided to pregnant women with COVID-19. Good clinical outcomes resulted for all mothers and babies except one female neonate who tested positive for COVID-19 and passed away on the fourth day after birth. Available records showed that the baby suffered from seizures, and her ABG showed an acidotic picture. CRP, electrolytes, and blood counts were within normal limits.

## Discussion

On April 30, 2021, India witnessed an unprecedented peak in incident COVID-19 cases and became the first country in the world to register over 400,000 infections in a single day [10]. The rise in case counts was grave enough that the United States issued a notice for “Suspension and Limitation on Entry” of persons who were in India during the prior two weeks [11]. This was also the day that our hospital faced a crisis in admissions with 10 referrals in one day. The trendline of deaths showed a corresponding spike. Many of the patients referred to our hospital during this crisis were in a moribund state, and possibly as a result, 12 of the 24 patients admitted during the week starting April 29, died within three days of admission. This may explain the dip in week 3 of the ALOS graph observed in Figure 1. As seen in Table 3, very few patients had a traceable source of infection indicating established community transmission by this stage of the pandemic. Garg et al. have reported evidence pointing to community transmission of the Omicron variant of COVID-19 (B1.1.529) in Delhi [12]. Sobagaiah et al. demonstrated an overall COVID-19 seropositivity rate of 20.3% among adults aged above 18 years in the slums of Bangalore during mid-April 2021 [13]. This is in contrast to findings from the first wave in 2020, when a social network analysis of COVID-19 transmission in Karnataka by Saraswathi et al. revealed that a limited number of source cases had infected close to 60% of all target cases, indicating negligible community transmission [14]. Thus, the results of our study, in conjunction with findings from other authors, shed light on the evolution of transmission characteristics between the first and second waves in Karnataka. The proportion of diabetics (35%) and hypertensives (33%) was high in our study. Furthermore, we noted that more than 80% of the patients had elevated blood glucose levels during the course of their illness. This may have been due to the corticosteroids administered to all but two patients. It may also reflect the pathophysiology of the body’s reaction to infection with COVID-19. Multiple authors have reported that increased blood glucose in COVID-19 patients is associated with the severity of progression and higher mortality [15-17].

Cost of hospitalization and treatment

This hospital was designated by the government as a referral center for tertiary COVID-19 care for the duration of the Delta wave. All regular services were suspended by government mandate, and the hospital was not permitted to admit any private patients. Patients showing symptoms suggestive of COVID-19 were triaged at government primary and secondary care facilities, and referrals were issued to only the most critical patients to ensure equity of care, and resources available to those most in need. All admitted patients were treated free of charge. Costs of hospitalization, treatment (including the cost of the antiviral drug Remdesivir), and final rites were borne by the Government of Karnataka. A cashless system was instituted, and the hospital was permitted to raise claims with Suvarna Arogya Suraksha Trust (SAST), [18] which is the agency in charge of implementing the Government of India’s flagship community health insurance program, Ayushman Bharat. SAST facilitates the processing of preauthorization and claims disbursements for Arogya Karnataka, the state arm of Ayushman Bharat. The Government of Karnataka created a special provision for free treatment of all COVID-19 patients who required hospitalization with sequential reimbursement bands for progressing levels of required care (general ward, ward with oxygen support, ICU without ventilation, ICU with ventilation). Claims were reimbursed at the rate of INR 9,750 per day for ICU without ventilation, and INR 11,500 per day for ICU with ventilation [19]. Remdesivir was procured by the hospital daily according to the number of vials required and dispensed from designated government pharmacies with authentication checks involving documentation of all prescriptions and return of used vials. These stringent checks were instituted due to reports of privateering and price gouging as remdesivir came to be perceived as a miracle cure and a desperate need by the general public [20]. In the event of death, the district health authorities were informed in writing, and the local (Taluk) health officials coordinated the disposal of mortal remains, including dispatching a designated ambulance to receive the body and transport it to the cemetery, and provision personal protective equipment (PPE) for two family members.

Limitations

This study was based on death audit reports generated from case sheets written by individual clinicians. Due to the unprecedented logistic pressures of the delta wave, the documentation was not uniform, and the researchers were unable to access complete laboratory records for all cases. Moreover, as postmortem examination of COVID-19 patients was not permitted, histopathological variables could not be studied or reported.

## Conclusions

This study reported the clinical and laboratory characteristics of 91 adult COVID-19 mortality cases at a teaching hospital that was repurposed into a COVID-19 referral treatment unit under government orders during April-July 2021, at the peak of the Delta wave in Karnataka. The age-sex distribution of patients in this study was concordant with the typical clinicodemographic picture of COVID-19. We noted a high proportion of patients with comorbidities diabetes mellitus and hypertension. While inflammatory indicators, such as WBC count, CRP, and d-dimer, were raised in many patients, our most remarkable finding was the high frequency of hyperglycemia (seen in more than four-fifths of the patients). Further study is indicated to explore the clinicopathological associations of glycemic dysregulation in COVID-19, and as a corollary, it is advisable to observe the survivor’s cohort for long-term incidence of diabetes mellitus. We expect that the findings of our study would contribute toward enhancing the understanding of the clinical correlates and progression of COVID-19 and may guide triage and treatment decisions in patients requiring intensive care if there are further waves of this novel pandemic or outbreaks of other novel respiratory viruses.
